# 

**Published:** 1858-06

**Authors:** 


VOL. IV.] [NUMBER 4.

APRIL, MAY AND JUNE.

1858

### IOWA MEDICAL JOURNAL.

CONDUCTED BY THE FACULTY OF THE

### MEDICAL DEPARTMENT

OF THE

### IOWA STATE UNIVERSITY.

PUBLISHED QUARTERLY.

TERMS—TWO DOLLARS, INVARIABLY IN ADVANCE

EDITORS:

J. C. HUGHES, M. D.,

Prof. of Surgery in the Medical Department of the Iowa University.

WELLS R. MARSH, M. D.,

Prof. of Chemistry and Toxicology in Medical Department of Iowa University.

KEOKUK, IOWA:

POST BOOK AND JOB OFFICE PRINT, 4TH BET. MAIN & JOHNSON STS.

1858.

